# A Mobile Just-in-Time Adaptive Intervention for Smoking Cessation: Pilot Randomized Controlled Trial

**DOI:** 10.2196/16907

**Published:** 2020-03-09

**Authors:** Emily T Hébert, Chaelin K Ra, Adam C Alexander, Angela Helt, Rachel Moisiuc, Darla E Kendzor, Damon J Vidrine, Rachel K Funk-Lawler, Michael S Businelle

**Affiliations:** 1 Oklahoma Tobacco Research Center Stephenson Cancer Center University of Oklahoma Health Sciences Center Oklahoma City, OK United States; 2 Moffitt Cancer Center Tampa, FL United States; 3 Department of Psychiatry and Behavioral Sciences Stephenson Cancer Center University of Oklahoma Health Sciences Center Oklahoma City, OK United States

**Keywords:** smartphones, mobile phones, smoking cessation, just-in-time adaptive intervention, mHealth

## Abstract

**Background:**

Smartphone apps for smoking cessation could offer easily accessible, highly tailored, intensive interventions at a fraction of the cost of traditional counseling. Although there are hundreds of publicly available smoking cessation apps, few have been empirically evaluated using a randomized controlled trial (RCT) design. The Smart-Treatment (Smart-T2) app is a just-in-time adaptive intervention that uses ecological momentary assessments (EMAs) to assess the risk for imminent smoking lapse and tailors treatment messages based on the risk of lapse and reported symptoms.

**Objective:**

This 3-armed pilot RCT aimed to determine the feasibility and preliminary efficacy of an automated smartphone-based smoking cessation intervention (Smart-T2) relative to standard in-person smoking cessation clinic care and the National Cancer Institute’s free smoking cessation app, QuitGuide.

**Methods:**

Adult smokers who attended a clinic-based tobacco cessation program were randomized into groups and followed for 13 weeks (1 week prequitting through 12 weeks postquitting). All study participants received nicotine patches and gum and were asked to complete EMAs five times a day on study-provided smartphones for 5 weeks. Participants in the Smart-T2 group received tailored treatment messages after the completion of each EMA. Both Smart-T2 and QuitGuide apps offer on-demand smoking cessation treatment.

**Results:**

Of 81 participants, 41 (50%) were women and 55 (68%) were white. On average, participants were aged 49.6 years and smoked 22.4 cigarettes per day at baseline. A total of 17% (14/81) of participants were biochemically confirmed 7-day point prevalence abstinent at 12 weeks postquitting (Smart-T2: 6/27, 22%, QuitGuide: 4/27, 15%, and usual care: 4/27, 15%), with no significant differences across groups (*P*>.05). Participants in the Smart-T2 group rated the app positively, with most participants agreeing that they can rely on the app to help them quit smoking, and endorsed the belief that the app would help them stay quit, and these responses were not significantly different from the ratings given by participants in the usual care group.

**Conclusions:**

Dynamic smartphone apps that tailor intervention content in real time may increase user engagement and exposure to treatment-related materials. The results of this pilot RCT suggest that smartphone-based smoking cessation treatments may be capable of providing similar outcomes to traditional, in-person counseling.

**Trial Registration:**

ClinicalTrials.gov NCT02930200; https://clinicaltrials.gov/show/NCT02930200

## Introduction

### Background

Although a majority of cigarette smokers are interested in quitting, very few use evidence-based cessation treatments [1]. Best practice guidelines for treating tobacco use and dependence suggest that a combination of counseling and medication is most effective for smoking cessation [2]; however, only 25% of US adult cigarette smokers reported using nicotine patches or gum during their most recent quit attempt, and only 15% sought help from a doctor or other health professional [3]. Smokers have reported a number of barriers to accessing tobacco cessation counseling, including the lack of time, transportation issues, and cost [4,5]. These barriers may be even more burdensome among individuals of a lower socioeconomic status (SES), who have higher rates of tobacco use and are less likely to quit despite similar numbers of quit attempts as those of a higher SES [6,7]. Therefore, improving access to smoking cessation interventions is an important step toward reducing smoking-related health disparities.

Mobile technology has enormous potential to overcome many of the barriers that have hampered the use of other empirically supported smoking cessation treatments among lower SES individuals [8,9]. Smartphone ownership is widespread; 81% of US adults overall and 71% of adults with annual household incomes less than US $30,000 reported owning a smartphone in 2019 [10]. Smartphone apps could offer easily accessible, highly tailored, intensive interventions at a fraction of the cost of traditional smoking cessation counseling [11]. A recent systematic review found that technology-based cessation interventions increased cessation rates compared with standard self-help treatments and produced comparable cessation outcomes among disadvantaged and nondisadvantaged groups [12].

Although there are hundreds of publicly available smoking cessation apps, a few have been empirically evaluated using a randomized controlled trial (RCT) design. In a content analysis of 252 available iPhone and Android apps for smoking cessation, Abroms et al [13] found that very few apps adhered to proven strategies for smoking cessation (eg, suggesting the use of effective medications, connecting to quit lines or clinics) [13]. QuitGuide is a free smartphone app developed by the Tobacco Control Research Branch at the National Cancer Institute (NCI) and based on content from Smokefree.gov [14]. Unlike most available apps, QuitGuide’s content adheres to established clinical practice guidelines [2] and includes features such as motivational messages to encourage users to make a quit attempt and detailed information about medications. To date, only one study has examined the efficacy of the QuitGuide app. Bricker et al [15] compared QuitGuide with a smartphone-delivered Acceptance and Commitment Therapy–based app (SmartQuit) in an RCT. The overall self-reported quit rate for participants who were randomized to QuitGuide was 8% compared with 13% for SmartQuit. Thus, there appears to be much room for improvement in phone-based treatments.

In addition to its ability to expand the reach of smoking cessation interventions, mobile technology also allows researchers to examine the dynamic nature of smoking relapse in greater detail than previously possible. Ecological momentary assessment (EMA), in which mobile devices are used to capture moment to moment experiences, allows for the measurement of phenomena in real time within natural settings [16,17]. Using EMA, it is possible to understand the patterns of affect, environment, and social context that individuals experience when undergoing a quit attempt [18]. Although EMA utilizes self-reports, recall bias is greatly reduced by the frequency of measurement, and data are collected in natural contexts rather than laboratory-based settings [19]. A number of factors and cues have been found to be associated with smoking lapse, including urge to smoke [20,21], proximity to others smoking [22], proximity to tobacco retail outlets [23,24], and stress [25-27]. Furthermore, real-time reports of smoking lapse contexts suggest that most lapse episodes occur within minutes of the onset of a craving [28].

### Just-in-Time Adaptive Interventions

A recent model for addressing dynamic health behaviors such as smoking lapse is the just-in-time adaptive intervention (JITAI) [29]. JITAIs aim to address moments of vulnerability for unhealthy behaviors (such as high-risk situations) by providing support in real time through mobile technology [30]. JITAIs have been used to target a wide variety of health behaviors, including physical activity [31,32], eating behavior [33], and substance use [34,35]. Although JITAIs for smoking cessation are relatively new, a few studies have shown initial promise. McClure et al [36] found that a mobile intervention that combined self-help content and adaptively tailored advice for managing medication side effects and nicotine withdrawal symptoms was feasible and acceptable among a group of smokers who were ready to quit. Naughton et al [37] demonstrated the feasibility of using geolocation data to trigger support messages to prevent smoking. Using EMA data from smokers undergoing a quit attempt, Businelle et al [26] created a smoking lapse risk estimator that identified 80% of all smoking lapses within 4 hours of the lapse. The algorithm was used in a follow-up study to deliver tailored messages based on a person’s momentary risk for smoking lapse, and it was found that urges to smoke and cigarette availability were significantly reduced when tailored urge messages were delivered by the app compared with instances where other types of messages were delivered [38]. Although these JITAIs show great potential for providing widely accessible, innovative treatment for smoking cessation, most JITAIs remain untested. The purpose of this study was to compare, in a pilot RCT, the feasibility and preliminary effectiveness of a smartphone-delivered JITAI for smoking cessation (Smart-Treatment; Smart-T2) with the NCI QuitGuide app and usual care in-person tobacco cessation treatment.

## Methods

### Participants and Procedure

Individuals were screened for eligibility following a provider referral or self-referral to the Tobacco Treatment Research Program (TTRP), which is located at the University of Oklahoma Health Sciences Center (OUHSC) campus in Oklahoma City. The TTRP offers free tobacco cessation counseling and pharmacotherapy to the public and facilitates the recruitment, screening, and enrollment of participants into research studies. Referrals are received through the electronic medical record, and via phone, the internet, fax, and word of mouth. Individuals were eligible to participate if they (1) demonstrated an English literacy level greater than the sixth grade, (2) were willing to quit smoking 7 days from their first visit, (3) were ≥18 years of age, (4) had an expired carbon monoxide (CO) level >7 ppm suggestive of current smoking, (5) reported smoking ≥5 cigarettes per day, (6) were willing and able to attend four in-person assessment sessions, and (7) had no contraindications for over-the-counter nicotine replacement therapy (NRT; ie, uncontrolled blood pressure, myocardial infarction within the past 2 weeks, or current pregnancy or plans to become pregnant during the study period). Participants were informed that the study purpose was to compare three smoking cessation treatment approaches and were provided with a detailed outline of study procedures, and written informed consent was obtained.

The study procedure was approved by the Institutional Review Board at the OUHSC. Data collection took place between May 2017 and October 2018. Participants were followed for 13 weeks (1 week prequitting through 12 weeks postquitting) and completed in-person assessments at baseline, on the quit date (1 week after baseline), and at 4- and 12-week postquit visits. All participants were provided with a smartphone (Samsung Galaxy On5) at the baseline visit, were trained to use their assigned app by the study staff, and were asked to carry the phone with them at all times. Study smartphones were used to prompt and deliver EMAs and included (1) a Call Staff function/button that automatically called study staff when/if participants had problems with the phone and (2) a Payment function/button that enabled participants to track their current level of EMA compliance and level of compensation. All data collected through the smartphone app were deidentified and encrypted. Participants were prompted to complete EMAs five times per day (four random assessments and one daily diary) for 5 weeks (1 week prequitting and 4 weeks postquitting). During the EMA period, participants were also asked to self-initiate EMAs when they had an urge to smoke or had already smoked.

Participants were compensated for attending in-person visits and for completing prompted EMAs (ie, daily diary and random EMAs). Specifically, participants received a US $30 gift card for attending and completing each of the first three postquit visits (ie, baseline, quit date, and 4 weeks) and US $50 for completing the 12-week postquit visit. At the 4-week postquit visit, participants received additional compensation based on the percentage of random and daily diary EMAs that they completed. Specifically, those who completed 50% to 74% of all prompted EMAs over the 5-week EMA period received US $50 in gift cards, those who completed 75% to 89% of prompted EMAs received US $100, and those who completed 90% or more of prompted EMAs received US $150. Participants were not compensated for completing self-initiated urge or smoking reports.

### Treatment Groups

At baseline, participants were randomized into one of the following treatment groups: (1) Smart-T2 phone-based automated smoking cessation treatment, (2) NCI QuitGuide app, or (3) usual tobacco cessation clinic care (TTRP) using a simple computer-generated randomization scheme. All participants were provided with a smartphone preloaded with their assigned smoking cessation app and/or the EMA app for 5 weeks. In addition, all participants received a 2-week supply of over-the-counter NRT (ie, patches and gum) for the initial postquit period.

#### Smart-Treatment

**Figure 1:**
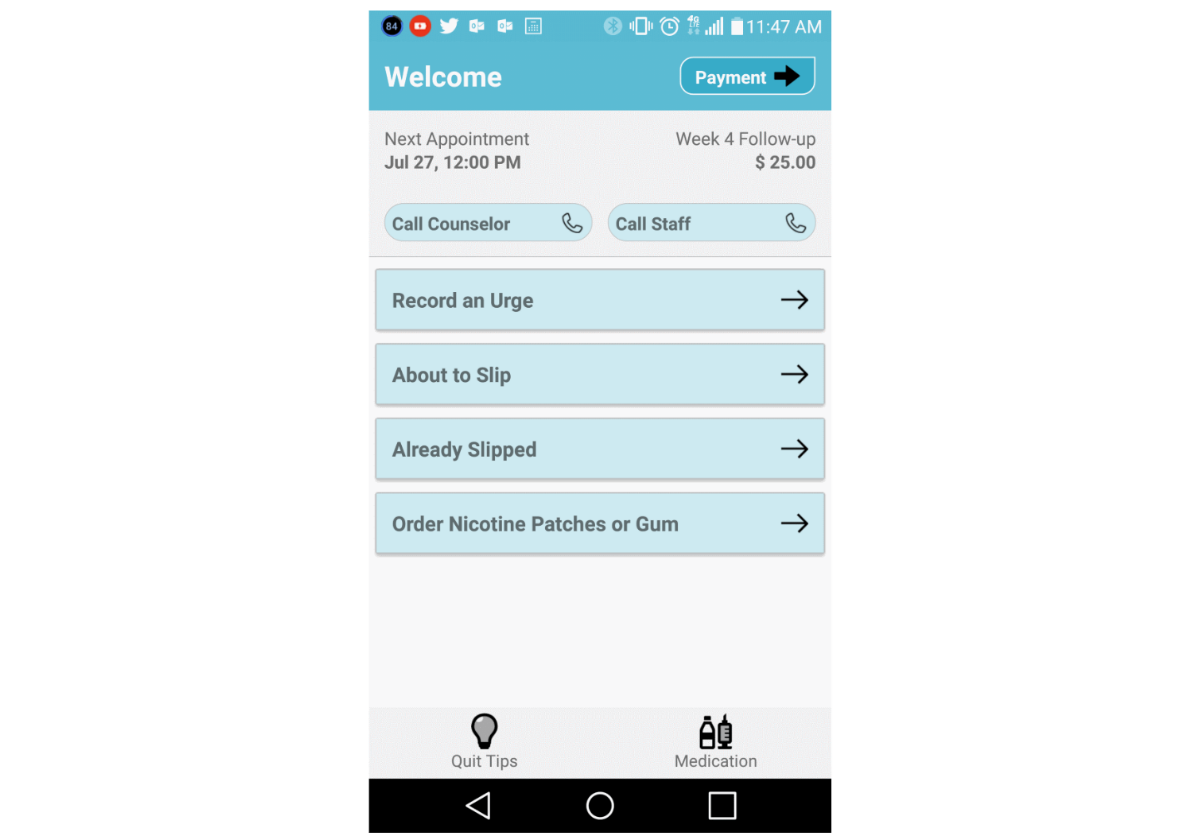
The Smart-Treatment app home screen.

The Smart-T2 app has been described in detail elsewhere [39]. Briefly, Smart-T2 is a multicomponent adjunctive smoking cessation app featuring (1) an algorithm that evaluates the current risk of smoking lapse based on EMA responses and pushes tailored messages to help participants cope, (2) a “Quit Tips” button offering cessation advice, coping strategies, and quitting benefits, (3) a “Medications” button offering information about smoking cessation medications, (4) a “Phone a Counselor” button that calls the free Oklahoma Tobacco Help Line, (5) daily treatment messages (eg, your quit date is tomorrow), and (6) a button to request additional NRT through the EMA app home screen (Figure 1).

Risk of smoking lapse was estimated in real time using a weighted lapse risk estimation formula developed by Businelle et al [26]. The formula included six variables shown to be associated with lapse, including urge to smoke, stress, recent alcohol consumption, interaction with someone smoking, motivation to quit, and cigarette availability. These lapse risk factors were weighted based on their ability to discriminate moments of high risk for lapse from moments of low risk for lapse (described in detail in a study by Businelle et al [26]). Intervention messages were delivered at the completion of every EMA. During the prequit period, participants received messages that aimed to prepare them for their upcoming quit attempt. During the 4-week postquit period, participants received automated, individually tailored messages based on their current level of risk for imminent smoking lapse and the presence of lapse triggers. When EMA responses indicated low risk for imminent smoking lapse, messages focused on maintaining abstinence motivation and general cessation advice. When EMA responses indicated high risk for imminent smoking lapse or the participant already smoked that day or the day before, or the participant indicated on their first daily assessment that they had a greater than 25% chance of smoking that day, tailored messages focused on ways to cope with current lapse risk symptoms (ie, reported during the current EMA) and were tailored to the highest rate of 4 current lapse triggers (ie, stress, smoking urge, easy access to cigarettes, and low motivation to quit). In addition, when EMA responses indicated a high risk of imminent lapse, participants received a message to chew a piece of nicotine gum to reduce their risk for lapse. When a participant indicated that they lapsed and were no longer interested in quitting smoking, messages focused on treating the lapse as a learning experience and supported a return to abstinence.

#### QuitGuide

The NCI’s QuitGuide app is a free smartphone app that is available through the Smokefree.gov website [14]. The QuitGuide app aims to help smokers understand their smoking patterns and develop the skills needed to quit smoking. Participants can track cravings, mood, smoking triggers, and motivations for quitting. Participants can also access features that provide information about the health consequences of smoking and quitting, smoking cessation medications, ways to handle urges to smoke, developing a multicomponent smoking cessation plan, and coping with lapse. Finally, participants have the option to schedule automated messages to be delivered when they are in a specific location or at a specific time.

#### Usual Care

Usual tobacco cessation treatment in the TTRP was based on established clinical practice guidelines [2] and included six weekly individual counseling sessions from 1 week before the quit date through 4 weeks postquit date. A total of five unique topics were discussed based on their relevance to the participant at each visit: (1) the impact of tobacco on health/benefits of quitting, (2) stress management strategies, (3) making positive lifestyle changes, (4) developing coping skills, and (5) relapse prevention. The counselor checked in with participants each week about the difficulties and successes they experienced and helped to plan for anticipated challenging situations. Advice and support were provided as needed. In-person counseling was provided at baseline, on the quit date, and at 4-week postquit visits, and all other counseling visits were completed either in-person or via telephone.

### Measures

At baseline, participants answered demographic questions including questions on age, sex, race/ethnicity, and smoking history. On the quit date, participants were asked if they smoked “even a puff” since 10:00 PM on the night before their quit date visit. At each in-person visit following the scheduled quit date, participants were asked if they smoked “even a puff” during the past 7 days. Abstinence was verified via expired CO at each visit using a Vitalograph CO monitor. Self-reported abstinence over the specified time period and a CO reading below 6 ppm (10 ppm on the quit date) were required to be considered abstinent. Participants who did not provide biochemical confirmation of abstinence (eg, they did not attend the visit) were considered smoking. On the quit date and at 4-week postquit visits, participants also answered questions to evaluate their satisfaction with their smoking cessation counselor or assigned smoking cessation smartphone apps.

During each EMA, participants answered questions about psychological, social, and environmental factors including stress, urge to smoke, cigarette availability, motivation to quit, recent alcohol consumption, and interaction with someone smoking. Participants were asked to rate on a scale from 1 (strongly disagree) to 5 (strongly agree) if they had an urge to smoke, if they felt stressed, how easily cigarettes were available to them (1 [not at all] to 5 [easily available]), if they were motivated to avoid smoking (1 [strongly agree] to 5 [strongly disagree]), whether they were interacting with anyone who was smoking (yes/no), and whether they drank alcohol in the last hour (yes/no). Participants were also asked to report the likelihood that they would “smoke between now and the end of the day.” During daily diary EMAs, participants were asked to report on behaviors and their environment the prior day (eg, how many pieces of nicotine gum they chewed [0 to 8 or more], how many hours that they wore a nicotine patch [I did not wear the patch at all to I wore the patch for at least 22-24 hours], and the number of alcoholic drinks [0 to 8 or more]). Nicotine patch wear time was recoded as a continuous variable by selecting the middle time point of each category used to indicate the period of daily patch wear time (ie, “I did not wear it at all”=0 hours, “Less than 3 hours”=2 hours, “4-6 hours”=5 hours, etc).

### Data Analysis

Descriptive statistics were used to summarize participant demographics and engagement with the smartphone app. Comparisons between groups were made using chi-square tests or analyses of variance with a Fisher least significant difference post hoc test, as appropriate. All analyses were conducted in IBM SPSS version 26.

## Results

### Participants

**Figure 2:**
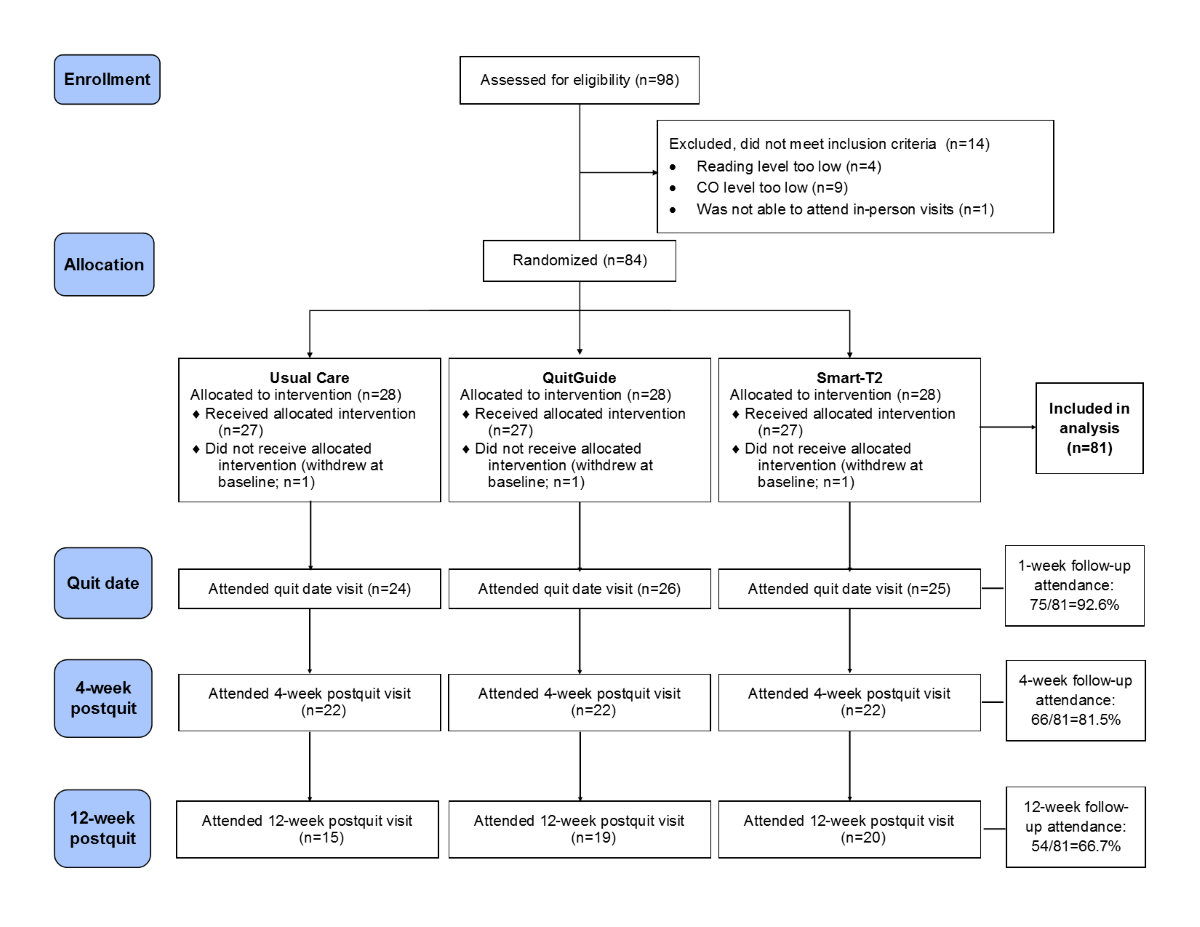
Consolidated Standards of Reporting Trials diagram. CO: carbon monoxide.

A total of 98 individuals were assessed for eligibility. Of those, 84 were eligible and consented to participate in the study. Subsequently, 3 individuals dropped out of the study before the baseline visit was completed, and thus all analyses included the remaining 81 participants (Figure 2). Participants were 51% (41/81) women, were mostly white (55/81, 68%), were on average aged 49.6 years, and smoked, on average, 22.4 cigarettes per day at baseline (Table 1). Over the course of the study, 27 participants either withdrew (ie, discontinued participation in the study) or were lost to follow-up (Figure 2). The average age was significantly different across treatment groups (*P*<.007); however, there were no other significant differences in other demographic variables, withdrawal, or loss to follow-up across groups (all *P* values >.05).

**Table 1:** Participant demographics.

Characteristic			Total (N=81)		Usual care (n=27)		QuitGuide (n=27)		Smart-Treatment (n=27)
Age (years), mean (SD)			49.6 (11.9)		51.3 (10.1)^a^		44.0 (12.6)^a^		53.6 (11.1)^a^
Gender (female), n (%)			41 (50)		12 (44)		16 (59)		13 (48)
**Race, n (%)**									
	White	55 (67)		18 (66)		19 (70)		18 (66)	
	Black	14 (17)		6 (22)		6 (22)		2 (7)	
	Other	12 (14)		3 (11)		2 (7)		7 (25)	
Annual household income (<US $30,000), n (%)			40 (49)		15 (55)		14 (51)		11 (40)
Cigarettes smoked per day at baseline, mean (SD)			22.4 (12.6)		21.7 (13.0)		22.9 (14.9)		22.7 (10.0)
Withdrew or lost to follow-up, n (%)			27 (33)		12 (44)		8 (29)		7 (25)

^a^Values 51.3 (10.1) and 53.6 (11.1) are not significantly different from each other but the value 44.0 (12.6) is significantly different from both at the *P*<.05 level.

### Ecological Momentary Assessment Completion and Treatment Engagement

Over the study period, participants completed a total of 2384 prompted daily diary and 7688 prompted random EMAs, with an overall compliance rate of 84.0%. Participants self-initiated 3253 EMAs, including reporting cigarette smoking in the prequit period, and lapse and urge to smoke in the postquit period. Compliance with prompted EMAs did not significantly differ across treatment groups. Most phones (72/81, 89%) were returned undamaged. In the usual care group, participants received an average of 3.8 counseling sessions (range 1-6 sessions).

#### Smart-Treatment

**Figure 3:**
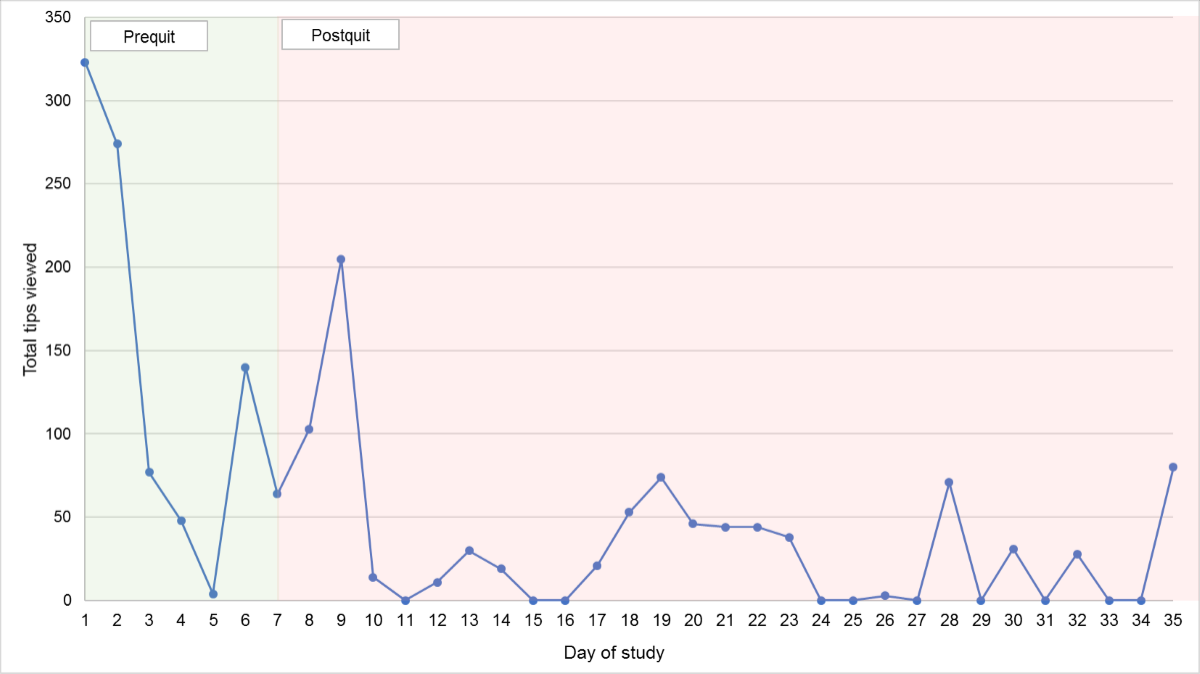
The distribution of on-demand tips accessed over time.

Among the 27 participants in the Smart-T2 group, 14 participants (52%) accessed the on-demand medication tips, and 20 (74%) participants accessed the on-demand quit tips. Once a participant selected a specific category of message, they could click “next” to view multiple messages within that category. The most frequently selected tip types were “Coping with Others Smoking” and “Harms of Smoking” (selected an average of 2.0 times during the intervention period), followed by “Coping with Mood” (selected an average of 1.8 times), and “Medication: Nicotine Gum” (selected an average of 1.5 times). On average, participants viewed the most messages within “Coping with Others Smoking” (mean 60.0 messages, SD 7.1), “General Quitting Advice” (mean 58.3 messages, SD 50.2), and “Harms of Smoking” categories (mean 42.0 messages, SD 26.1). The number of on-demand tips accessed varied over time (Figure 3) with the majority of tips viewed on days 1 and 2 of the study and declining on day 3. There was a sharp increase in the number of tips viewed on day 6 (the day before the scheduled quit day) as well as day 9 (2 days into the quit attempt).

**Figure 4:**
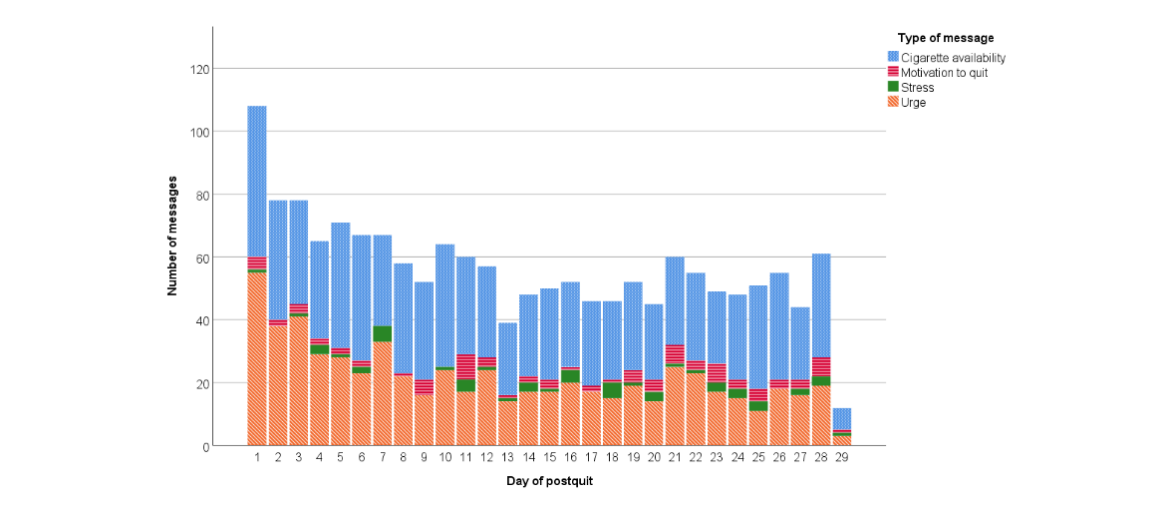
The distribution of high-risk messages over time.

A total of 66% (18/27) of participants in the Smart-T2 group used the Order NRT button to request a refill of NRT and 40% (11/27) of participants used the Call counselor button to reach the Oklahoma Tobacco Cessation Helpline, for an average of 2.2 button pushes during the 5-week EMA period. During the course of the intervention, a total of 3873 messages were delivered to participants in the Smart-T2 group. Each Smart-T2 participant received 145 treatment messages on average during the study. Of the high-risk tailored messages delivered, a majority (869/1638, 53.05%) were related to easy cigarette availability, followed by urge to smoke (630/1638, 38.46%), motivation to quit (85/1638, 5.19%), and stress (54/1638, 3.30%). Figure 4 shows the distribution of tailored messages over the course of the postquit period.

#### QuitGuide

Among the participants in the QuitGuide treatment group, 78% (21/27) of participants opened the app at an average of 9.9 times (SD 7.4) and interacted with the application for an average of 10.6 days (SD 4.8; range 2-20 days) during the 5-week EMA period. Within each day interacting with QuitGuide, participants completed an average of 5.5 actions (ie, a unique button press that initiates an app feature, eg, opening the journal; SD 5.9; range 1-32 actions). Over the study period, 59% (16/27) of participants used the Manage my Mood feature at an average of 3.0 times, 33% (9/27) of participants accessed the How to Quit feature at an average of 2.1 times, and 41% (11/27) of participants used the journal at an average of 1.7 times. Only 4 participants used the Location Help feature, which allowed them to tag a location in which they would receive a message to prevent lapse or to cope with craving to smoke. In addition, only 2 participants used the Share my Stats feature to share their progress on social media.

### Smoking Cessation Outcomes

A total of 26% (21/81) of participants were confirmed abstinent (ie, 7-day point prevalence, intent to treat) at 4 weeks postquitting (Smart-T2: 6/27, 22%, QuitGuide: 7/27, 26%, usual care: 8/27, 30%), and 17% (14/81) participants were confirmed abstinent at 12 weeks postquitting (Smart-T2: 6/27, 22%, QuitGuide: 4/27, 15%, usual care: 4/27, 15%). There were no significant differences in smoking abstinence across treatment groups at any time point.

### Nicotine Replacement Therapy Utilization

On each daily diary, participants reported the number of pieces of gum that they chewed and the number of hours that they wore a nicotine patch the previous day. On average, participants reported chewing 5.1 pieces of gum each day (SD 2.5; range 0-8 or more) and wearing a nicotine patch for an average of 19.4 hours each day (SD 5.6; range=0-23). There were no significant differences in the number of pieces of gum chewed or patch wear time across treatment groups. Within the Smart-T2 group, when the risk for lapse was high, participants also received a message stating, “Chewing a piece of nicotine gum right now may reduce your risk for smoking. Will you chew a piece of nicotine gum right now?” The nicotine gum message was delivered 861 times, 31.9 times per participant on average (SD 44.0). Participants responded “yes,” that they would chew a piece a gum in 65.2% of cases.

### Treatment Satisfaction

Across all three groups, a majority of participants agreed or strongly agreed with the statements, “I can rely on my (treatment) to provide guidance that will help me to quit smoking and stay quit” (46/65, 70.8%) and “I believe that my [treatment] will help me to quit smoking and stay quit” (45/65, 69.2%. The mean response on a scale from 1 (strongly disagree) to 5 (strongly agree) for both questions was significantly higher in the usual care group compared with the QuitGuide group, but not significantly different from the mean Smart-T2 group response (Table 2). Participants in the usual care group reported a significantly higher mean response agreeing with the statement, “I feel that my [treatment] provides smoking cessation treatment that is personalized to my specific needs,” compared with both the QuitGuide and Smart-T2 groups. Participants in both the usual care and Smart-T2 groups reported significantly higher average responses (ie, strongly agreeing, agreeing) to the statements, “My [treatment] knows how to help me quit smoking” and “I believe I can depend on my [treatment],” and reported significantly lower mean responses (ie, strongly disagreeing, disagreeing) to the question, “Do you find the smartphone application to be annoying?” (Table 2).

**Table 2:** Treatment satisfaction and app perceptions at week 4 postquit.

Treatment group		Value, mean (SD)	SE	*F* test (*df*)	*P* value
**I can rely on my treatment to provide guidance that will help me to quit smoking and stay quit**					.04
	Usual Care (n=21)	4.33^a^ (0.66)	0.14	3.34 (2,62)	
	QuitGuide (n=22)	3.59^b^ (1.14)	0.24	3.34 (2,62)	
	Smart-T2^a^ (n=22)	3.95^a,b^ (0.95)	0.20	3.34 (2,62)	
	Total (N=65)	3.95 (0.98)	0.12	3.34 (2,62)	
**I feel that my treatment provides smoking cessation treatment that is personalized to my specific needs**					.01
	Usual Care (n=21)	4.33^a^ (0.66)	0.14	5.45 (2,62)	
	QuitGuide (n=22)	3.59^b^ (1.05)	0.23	5.45 (2,62)	
	Smart-T2 (n=22)	3.55^b^ (0.86)	0.18	5.45 (2,62)	
	Total (N=65)	3.82 (0.93)	0.12	5.45 (2,62)	
**I believe that my treatment will help me to quit smoking and stay quit**					.04
	Usual Care (n=21)	4.33^a^ (0.80)	0.17	3.52 (2,62)	
	QuitGuide (n=22)	3.64^b^ (1.05)	0.22	3.52 (2,62)	
	Smart-T2 (n=22)	4.09^ab^ (0.75)	0.16	3.52 (2,62)	
	Total (N=65)	4.02 (0.91)	0.11	3.52 (2,62)	
**My treatment knows how to help me to quit smoking**					.02
	Usual Care (n=21)	4.29^a^ (0.78)	0.17	4.23 (2,62)	
	QuitGuide (n=22)	3.59^b^ (1.10)	0.23	4.23 (2,62)	
	Smart-T2 (n=22)	4.23^a^ (0.69)	0.15	4.23 (2,62)	
	Total (N=65)	4.03 (0.92)	0.11	4.23 (2,62)	
**I believe I can depend on my treatment**					<.001
	Usual Care (n=21)	4.33^a^ (0.80)	0.17	7.48 (2,62)	
	QuitGuide (n=22)	3.27^b^ (1.24)	0.27	7.48 (2,62)	
	Smart-T2 (n=22)	4.05^a^ (0.65)	0.14	7.48 (2,62)	
	Total (N=65)	3.88 (1.02)	0.13	7.48 (2,62)	
**Do you find the smartphone application to be annoying?**					.01
	Usual Care (n=22)	2.45^a^ (1.37)	0.29	5.11 (2,63)	
	QuitGuide (n=22)	3.41^b^ (1.40)	0.30	5.11 (2,63)	
	Smart-T2 (n=22)	2.23^a^ (1.11)	0.24	5.11 (2,63)	
	Total (N=66)	2.70 (1.38)	0.17	5.11 (2,63)	

^a^All scales rated from 1=strongly disagree to 5=strongly agree.

^b^Pairwise comparisons are indicated with a superscript. Values that do not share a letter are significantly different at the *P*<.05 level.

## Discussion

### Principal Findings

This study demonstrates the feasibility and acceptability of Smart-T2, a smartphone-based, JITAI for smoking cessation. Participants across all three treatment groups (ie, Smart-T2, QuitGuide, and usual care) were largely compliant with the EMA protocol, and a majority of participants in the Smart-T2 group engaged with on-demand treatment content and utilized the app to order additional NRT. Although the study was not powered to detect significant differences in smoking cessation outcomes or NRT use across the three treatment groups, the results of this pilot RCT suggest that smartphone-based smoking cessation treatments may be capable of providing similar outcomes to traditional, in-person counseling. Participants in the Smart-T2 group rated the app positively, with most participants agreeing that they can rely on the app to help them quit smoking and endorsed the belief that the app would help them stay quit, and these responses were not significantly different from the ratings given by participants in the usual care group.

### Treatment Engagement

Engagement with digital behavior change interventions has been defined as “the extent (e.g., amount, frequency, duration, depth) of usage and a subjective experience characterized by attention, interest, and affect” [40]. Research has demonstrated that engagement with smoking cessation apps is typically low [41]. For example, Zbikowski et al [41] tracked program utilization for an integrated phone and Web-based tobacco cessation program and found that of 11,143 participants, users logged into an app at an average of 1 to 2 times and completed an average of 2 to 2.5 counseling calls. In the Smart-T2 group, participants consistently engaged with the EMAs for the duration of the study period, and most participants engaged with the on-demand content at least once. In this study, engagement with the QuitGuide app was lower compared with the Bricker et al [15] trial wherein participants self-reported opening the app at an average of 15.2 times compared with an average of 9.9 times in this study. However, it is worth noting that participants assigned to the QuitGuide intervention arm in this study had to manage two separate apps (the QuitGuide app and the EMA app) to access intervention content and complete EMAs, whereas those in the Smart-T2 intervention were reminded about the on-demand content because the home screen was displayed at the completion of each EMA. In addition, participants in Bricker et al [15] self-reported the number of times they opened the app, whereas engagement statistics in this study were objectively recorded in the QuitGuide app and exported.

Within the Smart-T2 group, participants accessed the most messages within the “General Quitting Advice” and “Coping with Others Smoking” categories, on average. The fact that participants were drawn to the general quitting advice category may suggest that many participants were seeking general coping strategies to aid their cessation attempt. However, the high number of tips viewed from the “Coping with Others Smoking” category is consistent with the fact that a majority of automated, tailored messages delivered were related to easy cigarette availability. Over the course of the study, most on-demand tips were viewed within the first 2 days of the prequit period, and on the second and third days of the postquit period. This pattern may suggest that participants desire greater treatment content in the first few days after downloading an app and in the immediate period after the quit date, or that participants were initially curious about treatment content and then lost interest over time. Future interventions could examine if having tips or treatment content that becomes available over time is associated with a more consistent pattern of engagement for the duration of treatment compared with on-demand content that is available all at once.

### Treatment Satisfaction

Participant treatment satisfaction in this pilot study may also have implications for future mobile smoking cessation interventions. Participants in the Smart-T2 group and usual care groups found the EMA app significantly less “annoying” compared with those in the QuitGuide group. A possible explanation is that participants in the Smart-T2 group were receiving tailored treatment content at the end of each EMA, whereas participants in the QuitGuide group only received smoking cessation treatment through a separate app. Repeated surveys during the day can be burdensome; however, participants may find the surveys more useful if they know that they are driving the delivery of content that is specifically relevant to their current socioenvironmental context. Across a range of health behaviors, tailored treatments have been shown to be superior to the more commonly used “one-size-fits-all” treatment approach [42-45]. This study further illustrates that dynamically tailored content enhances participant engagement and may increase acceptability of smartphone-based cessation interventions.

It is promising that both app-based interventions (Smart-T2 and QuitGuide) performed at least as well as the traditional, in-person counseling in terms of response rates, loss to follow-up, participant perceptions of the treatment, and engagement. The patterns of engagement and the participant perceptions of the intervention are consistent with findings in the literature. For example, Oliver et al [46] surveyed 224 daily cigarette smokers and asked them to describe the utility of features within smartphone apps for smoking cessation. Features that were rated as most important included gain-framed messages such as “tells me how much my health is improving each day that I don’t smoke” as well as “develops a personalized quit plan for me” and “helps me track my stress and craving levels.” Participants in both the QuitGuide and the Smart-T2 intervention groups interacted most frequently with features that offered general quitting advice, as well as features that helped manage their stress and mood. Both the QuitGuide and the Smart-T2 intervention groups provided tailored treatment with smoking cessation advice with either a personalized quit plan (ie, QuitGuide), or specific coping strategies to deal with the smoking triggers reported by participants in the moment (ie, Smart-T2).

### Smoking Cessation Outcomes

Although there has been a proliferation of smartphone-based smoking cessation interventions, very few RCTs have been conducted to test the efficacy of these interventions. A recent review of mobile applications for the treatment of tobacco use and dependence [47] found that only four well-powered studies have tested efficacy or effectiveness of smoking cessation apps, with abstinence rates ranging from 0.9% to 12% at the end of the study [47]. A review of smoking cessation interventions for disadvantaged groups found a similar dearth of high-quality studies that have examined the effectiveness of technology-based smoking cessation among disadvantaged populations [12], yet there is preliminary evidence that they are effective at increasing quit rates at 1-, 3-, 6-, and 18-month follow-ups. The sample size of this pilot trial precludes the ability to make definitive conclusions about the effectiveness of this intervention; however, the current preliminary results of a 12-week biochemically confirmed quit rate of 22.2% in the Smart-T2 group and 14.8% in the QuitGuide group may suggest that combining nicotine therapy with a mobile app could improve quit rates.

To our knowledge, only one other study, Bricker et al [15], has examined the effectiveness of the QuitGuide app, with a self-reported 30-day point prevalence cessation rate of 8% at a 2-month follow-up. In contrast, 14.8% of participants in the QuitGuide group of this study were biochemically confirmed abstinent at 12 weeks postquitting. However, the studies differed in terms of the method of measuring abstinence (ie, self-reporting vs biochemical verification and completers only vs intent to treat) and in terms of treatment protocol (ie, QuitGuide participants in this study were provided with NRT); thus, it is unclear if these results will generalize to a larger, fully powered sample.

### Strengths and Limitations

The strength of this study is the RCT design, which allowed for a preliminary comparison of the Smart-T2 app with in-person smoking cessation treatment and the NCI QuitGuide app that adheres to many of the recommendations of the smoking cessation clinical practice guidelines [2]. This study also has several limitations. First, the Smart-T2 app did not save the duration of counseling calls that were initiated through the app. Thus, we were unable to determine if use of the “Call Counselor” button was representative of legitimate calls to the Oklahoma Tobacco Cessation Helpline. Second, this pilot trial had a small sample size, which precludes the ability to make definitive conclusions about the effectiveness of the interventions. A future study will compare the effectiveness of the Smart-T2 app and QuitGuide intervention in a larger trial [48].

### Future Directions

The Smart-T2 app and similar interventions may represent a potential way to address the substantial tobacco-related health disparities experienced by low SES smokers who may have limited access to in-person treatment. To our knowledge, no interventions to date besides our recent pilot work [39] have used EMAs to repeatedly assess current smoking lapse risk and automatically deliver tailored treatment content. This preliminary work indicates that smartphones may be used to deliver well-liked, automated, tailored, low burden, and easily accessible interventions to smokers seeking smoking cessation treatments.

## Appendix

Multimedia Appendix 1 CONSORT-EHEALTH Checklist (V 1.6.1).

## Abbreviations

CO: carbon monoxide
EMA: ecological momentary assessment
JITAI: just-in-time adaptive intervention
NCI: National Cancer Institute
NRT: nicotine replacement therapy
OUHSC: University of Oklahoma Health Sciences Center
RCT: randomized controlled trial
SES: socioeconomic status
Smart-T2: Smart-Treatment
TTRP: Tobacco Treatment Research Program
